# Hemorrhagic Complications of Paracentesis: A Systematic Review of the Literature

**DOI:** 10.1155/2014/985141

**Published:** 2014-12-17

**Authors:** Kaveh Sharzehi, Vishal Jain, Ammara Naveed, Ian Schreibman

**Affiliations:** ^1^Temple University Hospital, Philadelphia, PA 19140, USA; ^2^Coastal Gastroenterology Associates, Brick, NJ 08724, USA; ^3^Abington Memorial Hospital, Abington, PA 19001, USA; ^4^Penn State Milton S. Hershey Medical Center, Hershey, PA 17033, USA

## Abstract

*Introduction*. Large volume paracentesis is considered a safe procedure carrying minimal risk of complications and rarely causing morbidity or mortality. The most common complications of the procedure are ascitic fluid leakage, hemorrhage, infection, and perforation. The purpose of this study was to evaluate all hemorrhagic complications and their outcomes and to identify any common variables. *Methods*. A literature search for all reported hemorrhagic complications following paracentesis was conducted. A total of 61 patients were identified. Data of interest were extracted and analyzed. The primary outcome of the study was 30-day mortality, with secondary endpoints being achievement of hemostasis after intervention and mortality based on type of intervention. *Results*. 90% of the patients undergoing paracentesis had underlying cirrhosis. Three types of hemorrhagic complications were identified: abdominal wall hematomas (52%), hemoperitoneum (41%), and pseudoaneurysm (7%). Forty percent of the patients underwent either a surgical (35%) or an IR guided intervention (65%). Patients undergoing a surgical intervention had a significantly higher rate of mortality at day 30 compared to those undergoing IR intervention. *Conclusion*. Abdominal wall hematomas and hemoperitoneum are the most common hemorrhagic complications of paracentesis. Transcatheter coiling and embolization appear to be superior to both open and laparoscopic surgery in treatment of these complications.

## 1. Introduction

Abdominal paracentesis is a routine diagnostic and therapeutic procedure in patients with ascites [1]. Large volume paracentesis (LVP) involves removal of as much ascitic fluid as possible to relieve symptoms of a tense abdomen and dyspnea. Randomized controlled trials have shown that LVP is safer and more effective than therapy with diuretics for the treatment of tense ascites [2, 3]. Patients treated with LVP supported by plasma volume expansion have shortened hospitalization, better-preserved systemic hemodynamics, and renal function. Additional benefits include improvement in hepatic hemodynamics, decreased risk of developing spontaneous bacterial peritonitis, and less frequent hepatic encephalopathy. Thus, considerable evidence favors the use of LVP with albumin replacement as the preferred treatment of tense ascites [4].

LVP carries risk of complications. Studies have reported leakage of ascitic fluid, infection, bleeding, and bowel perforation following paracentesis. Mortality related to the procedure is rare but has been documented. Data has varied on the incidence of complications. Hemorrhagic complications of paracentesis are perhaps one of the most common immediate and late complications which are associated with morbidity and mortality. This group of complications has been conveniently attributed to the presence of coagulopathy and/or thrombocytopenia. Two large-scale studies have reported very low incidence of complication with LVP despite thrombocytopenia (mean platelet count of 50.4 × 10^3^/*μ*L) and coagulopathy (mean INR of 1.7 ± 0.46) [2, 4, 5]. On the other hand, there are multiple case reports and series of hemorrhagic and nonhemorrhagic complications of LVP published in the literature contradicting the results of other larger scale studies.

Death secondary to hemorrhage following paracentesis is a known complication and a few isolated cases have been reported in the literature [6–8]. The exact incidence of hemorrhagic complications after LVP in patients with cirrhosis and portal hypertension is unknown.

Practice guidelines from various societies such as the American Association for the Study of Liver Diseases (AASLD), the International Ascites Club, and the European Association for the Study of the Liver (EASL) do not consider paracentesis to be unsafe in the presence of marked thrombocytopenia or prolongation in the prothrombin time nor recommend correction of these parameters prior to paracentesis [3, 4, 9].

Since paracentesis has rare complications, there are no prospective studies or randomized trials addressing this issue and most published data are case reports or case series. There is no consensus on how to prevent these complications, nor are there recommendations on optimal management of them. Our goal was to review all hemorrhagic complications related to paracentesis reported in the literature, assess their outcome, and identify any common risk factors pertaining to their outcome.

## 2. Methods

This is a systematic review of all the published literature on hemorrhagic complications of paracentesis. For the purpose of this study we used multiple two-key word combinations to search the US National Library of Medicine National Institutes of Health (PubMed) and Google Scholar. The key words used for our search were “paracentesis,” “complication,” “hemorrhagic,” “bleeding,” “hemoperitoneum,” “retroperitoneal hematoma,” “inferior epigastric artery,” “aneurysm,” and “outcome.” We included letters, systematic reviews, review articles, case reports, classical articles, clinical conferences, and published abstracts from AASLD and the American College of Gastroenterology (ACG). One author reviewed all publications.

Any publication capable of providing individual level data on complications of paracentesis was reviewed in detail. We placed no time or language limitations. For publications where individual level data was not available the corresponding author was contacted for further information.

The variables of interest for this study were number of cases per publication, type of publication, demographic of subjects in each publication, etiology of liver disease, puncture site, type of operator, ultrasound guidance, pertinent laboratory information, type of intervention, intervention outcome, and patient outcome.

The primary outcome of the study was overall 30-day mortality. Secondary outcomes were achievement of hemostasis after intervention and mortality based on type of intervention.

All articles were reviewed by one author (KS) and the variables of interest were extracted on an Excel spreadsheet. We used an exact Fisher test (for 2 × 2 tables) to analyze dichotomous and nominal variables and one-way ANOVA was used for continuous variables. A *P* value of less than 0.05 was considered statistically significant. Statistical analysis was done using SPSS 18.

## 3. Results

We identified 31 publications that were pertinent to our clinical question. Despite multiple attempts contacting publisher and/or authors, we were unable to access 3 case reports published in Spain. We presented two case reports on hemorrhagic complication at the 2010 American College of Gastroenterology meeting that we included into our study [10].

From the 28 studies, which met our criteria, individual level data was not available in one study. From the remaining 27 [1, 6, 7, 10–32] studies we were able to extract individual level data on 61 cases that were included for analysis. Case reports, case series, cohort studies, and prospective studies contributed 24 patients, 29 patients, 7 patients, and 1 patient to this study, respectively. The oldest article dated back to 1951 and the most recent ones were published in 2011. The largest contribution came from the study by Pache and Bilodeau with 9 patients [20].

Table 1 summarizes the demographics of the subjects in our study.

Information on the etiology of cirrhosis was available in 38 out of 54 subjects (70%). The most common cause of cirrhosis was alcoholic, noted in 19 out of 38 subjects (50%). The etiology of ascites in the 6 noncirrhotic patients was tuberculosis, malignancy, acute liver failure (2 subjects), acute respiratory distress syndrome complicated with pleural effusion and ascites, and congestive heart failure.

Data on coagulopathy (defined as INR > 2), marked thrombocytopenia (defined as platelet count less than 50,000/*μ*L), and renal insufficiency (defined as GFR < 60 or creatinine > 1.2) were available in 46/61 (75%) patients, 36/61 (59%) patients, and 17/61 (27%) patients, respectively. 59% of patients had coagulopathy, 8% had marked thrombocytopenia, and 70% had some degree of renal insufficiency.

Nontrainee physicians performed the majority of paracentesis and the most common puncture site was in the lower quadrants. The summary of this data is shown in Table 2.

The type of hemorrhagic event was specified in 60 out of 61 patients (98%). Abdominal wall hematomas were most common hemorrhagic complication (52%), followed by hemoperitoneum in 41% and pseudoaneurysm in 7%.

46 out of 61 (75%) subjects received blood and blood products for severe hemorrhagic complications, 13 (21%) cases did not require blood transfusion, and one subject (4%) died due to severe hemorrhage before any intervention could be initiated.

24 out of 61 (40%) patients underwent a surgical or transcatheter intervention and 37 (60%) were managed conservatively. Type of intervention is summarized in Table 3. An attempt to identify the source of bleeding was made in 29 (47.5%) subjects; four of them were found after death. Close to 60% of the bleedings originated from inferior epigastric artery or one of its tributaries (Table 3).

The patient outcome was available in 60 out of 61 patients (97%). The 30-day mortality was 43.3% among all patients.  Table 4 shows patients outcome based on type of the intervention. We combined open and laparoscopic surgeries into “surgery” group and the various transcatheter embolization and coiling techniques into “interventional radiology” group. Better outcomes were seen in the transcatheter coiling and transcatheter embolization groups with 0% and 33% mortality, but these differences were not statically significant mainly due to their small numbers in each group.

The mortality was significantly higher in the surgical group (75%) compared with the interventional radiology group (25%) (Table 5). Given the small number of subjects in the groups we used Fisher's exact test which demonstrated a two-tailed *P* value of 0.0324. Univariate analysis of other predictors such as age, gender, type of hemorrhage, coagulopathy, thrombocytopenia, and real failure did show significant difference in outcome.

## 4. Discussion

Paracentesis is the most common therapy used by physicians for relief of symptoms associated with tense ascites from a variety of causes. The majority of these cases are performed in patients with end stage liver disease. Prior studies have shown that large volume paracentesis is a safe procedure, carrying approximately 1% risk of overall complications [11]. These complications include leakage of ascetic fluid, local infection, abdominal wall hematomas, intraperitoneal hemorrhage, and intestinal perforation.

The first published fatal hemorrhagic complication of paracentesis dates back to 1951 [7]. It was described as a rare complication of a “minor” procedure and it was speculated to be secondary to increased pressure in the collateral circulation and a “possible defect” in the blood clotting mechanism. Now over half a century later, this rare but potential lethal complication continues to occur.

Hemorrhagic complications of paracentesis can be broadly placed into 3 groups: abdominal wall hematoma, pseudoaneurysm, and hemoperitoneum. Our review demonstrated that abdominal wall hematoma is the most frequent hemorrhagic complication. Along with pseudoaneurysms of the inferior epigastric artery they constitute two-thirds of all hemorrhagic complications. Hemoperitoneum, which is usually the result of injury to a mesenteric varix, is responsible for close to one-third of these complications.

Serious hemorrhagic complications of paracentesis are seen both with large volume paracentesis and even with diagnostic paracentesis [16]. We did not have sufficient data to compare these groups.

Mallory and Schaefer [11] evaluated 242 consecutive diagnostic abdominal paracenteses in patients with liver disease and reported 4 patients with serious hemorrhagic complication (1.7%) which was significantly higher than previously published data. At that time they concluded that their higher complication rate could be related to selection bias and a “sicker” patient population.

Runyon [1] prospectively evaluated 229 abdominal paracenteses and reported one major complication (transfusion-requiring abdominal wall hematoma) (0.8%) and two minor complications (non-transfusion-requiring hematomas) (1.6%). They therefore concluded paracentesis to be a very safe procedure. However, the one patient who required blood transfusion sustained a variceal hemorrhage and succumbed to that. Although this does not appear to be directly related to the paracentesis, it demonstrates that “major bleeding” occurs in patients who are in a more advanced stage of their liver disease and their prognosis remains guarded regardless of the intervention performed.

In 1990, McVay and Toy [12] reviewed 608 procedures in 395 patients. The main goal of their study was to determine if untreated mild to moderate coagulopathy results in higher rates of hemorrhagic complications and if prophylactic plasma infusion affects the rate of hemorrhagic complication. The incidence of significant bleeding defined as more than 2-gram drop in Hgb was 3.1%, which was similar across different levels of coagulopathy. Among the patients with significant bleeding, only one patient required RBC transfusion (0.3%). The authors pointed out that they might have underestimated minor bleeding, since volume shifts after paracentesis can mask minor drop in Hgb. They concluded that for mild-moderate coagulopathy reversal of the coagulopathy is not indicated.

De Gottardi et al. in their prospective trial noticed higher rates of complications in patients with a higher Child-Pugh score [5]. Pache and Bilodeau noticed a similar trend in patients with a higher MELD and Child-Pugh score [20]. They also noticed a correlation between risk of hemorrhage and renal dysfunction rather than coagulopathy and thrombocytopenia. In our review renal dysfunction was also seen as the most prevalent metabolic derangement occurring in 70% of patients with bleeding, compared to coagulopathy and thrombocytopenia (59% and 8%, resp.). We did not observe a difference in frequency of these derangements among the subjects who survived or did not survive a hemorrhagic complication.

Traditionally, the midline has been considered avascular and safe area to attempt paracentesis; but multiple reports in the past demonstrated fatal and nonfatal hemorrhage from midline attempts [6–8]. These have been thought to be secondary to multiple periumbilical collaterals, which are often engorged and enlarged. In our review still 15% of paracenteses were performed in the midline.

Signs and symptoms of hemorrhage become evident from minutes to days after the procedure [13]. Bleeding at the time of or immediately after the procedure has been attributed to several factors including puncture of the superficial epigastric vessels resulting in abdominal wall hematomas or puncture of the intra-abdominal venous collaterals [6, 8]. Delayed postprocedure intra-abdominal hemorrhage is a rare complication. It has been reported up to 4 days after initial procedure [14]. A postulated mechanism for delayed hemorrhage is rapid decompression of splanchnic circulation due to release of intra-abdominal pressure. This could result in marked increase in portosystemic blood flow through collaterals with subsequent hemorrhage from collaterals. This has been the hypothesized mechanism for variceal bleeding after LVP reported by Liebowitz [33, 34]. Multiple reports have demonstrated the presence of large venous collaterals including recanalized umbilical veins. It may not be unreasonable to speculate the same here.

One of the first reviews on management of hemorrhagic complications was published by Arnold et al. in 1997 [14]. In a series of 4 patients, combinations of therapies were applied which included laparotomy and TIPS (transjugular intrahepatic portosystemic shunting) and TIPS in conjunction with embolization. Surgical management of acute spontaneous hemoperitoneum was described by Ben-Ari et al. [35] in 1995. In a series of 19 patients, who were treated using either ligation or portacaval shunting, 14 died postoperatively. Since these cases were spontaneous hemoperitoneum rather than postprocedural, we did not include them in our review, but the high mortality from surgery in their series (73%) is quite comparable with the results of our review (75%).

The use of transcatheter arterial embolization for spontaneous hemoperitoneum in the setting of ruptured hepatoma dates back to 1985 where few case reports were published [36]. One of the first large reviews on the use of transcatheter techniques was published by Hirai et al. In a series of 47 patients with ruptured hepatoma, 14 patients underwent embolization treatment with an improved median survival from 13 days in the supportive measures group to 98 days in the intervention group [37]. Even though this does not match our study population, it still demonstrates significant survival improvement compared to supportive care or surgical management.

More pertinent to our review, Lam et al. in 1998 published the first data on the use of transcatheter techniques for treatment of large symptomatic inferior epigastric artery pseudoaneurysms which occurred after paracentesis [15]. Patients who presented days to weeks after their paracentesis with large abdominal wall masses were treated successfully using this method and surgery (which was considered standard of care at that time) was avoided [15, 38].

Since then multiple case reports and case series have been published on the use and effectiveness of transcatheter techniques for abdominal bleeding. One of the larger case series on transcatheter treatment of hemoperitoneum is from Park et al. They reviewed twelve cases with massive hemoperitoneum, 6 of which underwent paracentesis in the setting of cirrhosis. Almost all of these patients had injury to the inferior epigastric artery [24]. In patients with ascites, the artery is laterally displaced due to distension and stretching of the abdominal wall; therefore it is more prone to injury during paracentesis [19]. In this case series, all patients were alive at one-month follow-up.

Sobkin et al. have published the largest case series on transcatheter embolization of inferior epigastric hemorrhage with 19 patients in the case series. 40% of these patients had bleeding related to paracentesis and their overall clinical success rate was 90%, defined as loss of extravasation after embolization [25].

Our analysis of these studies along with smaller case series and case reports shows the superiority of transcatheter approach compared to surgical management. We believe our study is the first one to highlight this difference even though the practice of management of hemorrhagic complications of paracentesis has already shifted in that direction. It is important to note that our study did not show any difference in 30-day mortality outcome based on analysis of other predictors such as age, gender, type of hemorrhage, and presence of coagulopathy, thrombocytopenia, and renal failure.

The strength of our study is the extensive review of the literature performed by the group. We avoided any date or language limitations. Except for 3 case reports from Spain we have been able to capture all other cases. To our knowledge this is the first review to assess the outcome and mortality of hemorrhagic complications of paracentesis and compare the mortality based on different types of intervention.

The main limitation to our study is the retrospective and heterogeneous nature of the data collected. Case series in which individual level data was not present were excluded. This heterogeneity and lack of data prohibited us to comment on other variables of interest such as presence of renal dysfunction, coagulopathy, and frequency of image-guided paracentesis and their relationship to hemorrhagic complications.

This review can guide a wide range of clinicians such as internists, gastroenterologists, hepatologists, interventional radiologists, and surgeons on the prompt diagnosis and management of these conditions.

## Figures and Tables

**Table 1:** Demographic information of the study subjects.

Variable		Number of valid cases
Age (mean ± standard deviation)	50.4 ± 12.6	51
Sex (male/female)	26/16	42
Presence of cirrhosis %	90%	60

**Table 2:** Summary of operator type and puncture site.

Operator		Nontrainee physician	Trainee physician	Nurse practitioner
*N* (%)		20 (60.6%)	10 (30.3%)	3 (9.1%)

Puncture site *N* (%)	Midline	Left lower quadrant	Right lower quadrant	Unspecified lower quadrant
	5 (14%)	14 (39%)	10 (28%)	7 (19%)

**Table 3:** Identified site of bleeding and type of intervention in subjects in hemorrhagic complication.

Type of intervention *N* (%)	Open or laparoscopic surgery		Transcatheter intervention	
	8 (33.3%)		16 (66.72%)	

Site of bleeding *N* (%)	Inferior epigastric artery	Mesenteric varix	Unidentified	Other
	17 (58.6%)	6 (20.7%)	3 (10.3%)	3 (10.3%)

**Table 4:** Subjects' 30-day outcome based on type of intervention.

30-day outcome	Type of intervention				
	*N* (%)				
	Conservative management	Surgery	Coiling	Embolization	Liver transplant
Dead	16 (44%)	5 (71%)	0 (0%)	4 (33%)	1 (100%)
Alive	20 (56%)	2 (29%)	4 (100%)	8 (67%)	0 (0%)

Total	36	7	4	12	1

**Table 5:** Comparison of subjects' 30-day outcome between surgical and interventional radiology groups.

Outcome	Intervention	
	(*N*)	
	Surgical group	Interventional radiology group
Dead	6	4
Alive	2	12
Mortality (%)	75%	25%
